# Dual Targeting of Endothelial and Cancer Cells Potentiates In Vitro Nanobody-Targeted Photodynamic Therapy

**DOI:** 10.3390/cancers12102732

**Published:** 2020-09-23

**Authors:** Vida Mashayekhi, Katerina T. Xenaki, Paul M.P. van Bergen en Henegouwen, Sabrina Oliveira

**Affiliations:** 1 Cell Biology, Neurobiology & Biophysics, Department of Biology, Faculty of Science, Utrecht University, 3584 CH Utrecht, The Netherlands; v.mashayekhi@uu.nl (V.M.); a.t.xenaki@uu.nl (K.T.X.); p.vanbergen@uu.nl (P.M.P.v.B.e.H.); 2 Pharmaceutics, Department of Pharmaceutical Sciences, Faculty of Science, Utrecht University, 3584 CG Utrecht, The Netherlands

**Keywords:** nanobody, VEGFR2, vascular targeted photodynamic therapy, combination therapy

## Abstract

**Simple Summary:**

Photodynamic therapy (PDT) eradicates tumors by the local activation of a photosensitizer with appropriate light. Although very promising, its clinical application is limited by the sub-optimal tumor specificity of the photosensitizer. Nanobody-targeted PDT was developed to improve specificity and efficacy of the treatment, using nanobodies that target the photosensitizer specifically to proteins overexpressed on cancer cells. Notably, intratumoral heterogeneity and/or low/moderate expression of some of these proteins hamper the efficacy of targeted PDT. To circumvent these, we explored the combination of cancer and endothelial cell targeting using nanobodies. For that, we developed nanobodies targeting mouse VEGFR2, which is mainly overexpressed on tumor vasculature, and we combined these with an EGFR targeted nanobody. Dual targeting of mouse endothelial and human cancer cells in a co-culture setup, using nanobody-photosensitizer conjugates, showed improved efficacy. In vivo follow up studies will reveal the full potential of this promising approach.

**Abstract:**

Photodynamic therapy (PDT) induces cell death through local light activation of a photosensitizer, although sub-optimal tumor specificity and side effects have hindered its clinical application. We introduced a new strategy named nanobody-targeted PDT in which photosensitizers are delivered to tumor cells by means of nanobodies. As efficacy of targeted PDT can be hampered by heterogeneity of target expression and/or moderate/low target expression levels, we explored the possibility of combined targeting of endothelial and cancer cells in vitro. We developed nanobodies binding to the mouse VEGFR2, which is overexpressed on tumor vasculature, and combined these with nanobodies specific for the cancer cell target EGFR. The nanobodies were conjugated to the photosensitizer IRDye700DX and specificity of the newly developed nanobodies was verified using several endothelial cell lines. The cytotoxicity of these conjugates was assessed in monocultures and in co-cultures with cancer cells, after illumination with an appropriate laser. The results show that the anti-VEGFR2 conjugates are specific and potent PDT agents. Nanobody-targeted PDT on co-culture of endothelial and cancer cells showed improved efficacy, when VEGFR2 and EGFR targeting nanobodies were applied simultaneously. Altogether, dual targeting of endothelial and cancer cells is a promising novel therapeutic strategy for more effective nanobody-targeted PDT.

## 1. Introduction

Photodynamic therapy (PDT) is a minimally invasive approach for cancer treatment. In this approach, three essential elements are needed to induce local cytotoxicity: a light-activatable photosensitizer (PS), light of a specific wavelength, and oxygen molecules. The activated PS can transfer energy to oxygen and subsequently form cytotoxic reactive oxygen species (ROS), among other reactive molecular species [1]. The produced reactive species destroy tumor cells, damage tumor vasculature, and induce immune response [2]. Currently, PDT is used in the clinic for the treatment of cancers such as bladder, skin, head and neck, and prostate [3,4,5]. Due to the hydrophobicity of most of the clinically approved PSs, site-specific light should be applied 2–4 days after the systemic administration of the PS in order to favor the retention of the PS in the tumor. This is mainly driven by the enhanced permeability and retention (EPR) effect [6] due to the unorganized structure of tumor vasculature, enabling therapeutics (mainly (lipo)protein associated PS) to extravasate into the extravascular space [7]. In addition, impaired-lymphatic drainage causes the macromolecules to be retained longer in the tumor [6]. Therefore, longer drug to light intervals are employed in order to preferably damage tumor tissue and, more importantly, reduce damage to normal tissues. Alternatively, applying light shortly after the administration of the PS (0–30 min) preferentially damages tumor-associated vessels. This therapeutic strategy has been named vascular targeted PDT (VTP) [8]. In this approach, which is driven passively, illumination of the PS mainly confined to the blood vessels causes vessel constriction, blood flow stasis, and thrombus formation. The vascular shutdown after VTP can block nutrients and oxygen supply to the tumor, resulting in necrosis and tumor regression [9]. The therapeutic benefit of VTP has been recently recognized in the clinic. The PS known as TOOKAD^®^ (Negma Lerads, Elancourt, Ile-De-France, France; Steba Biotech, Strasbourg, France) was approved in 2017 in Europe and Israel for the treatment of men with low-risk prostate cancer [10].

Although VTP and conventional PDT are already used in the clinic, in the last decades, efforts have been made to increase specificity and efficacy of the therapy. Next to the local and temporal control of light application, accumulation of the PS specifically and selectivity at the tumor tissue and tumor associated vasculature, can improve the efficacy of the treatment and further decrease side effects, such as photosensitivity and damage to the surrounding nerves and muscles. To this end, certain proteins which only express or are more abundant on tumor cells/vasculature have been targeted using different targeting moieties, such as peptides, antibodies or antibody fragments, and nanocarrier systems, to deliver the PS specifically and selectively to the tumor tissue/vasculature [11]. Nanobody-targeted PDT is one such approach, which was developed in our group. In this approach, PS molecules are specifically associated with tumor cells by means of nanobodies. Nanobodies (NBs) are the variable domain of heavy chain only antibodies that are naturally found in camelids and considered as the smallest antigen binding fragments [12]. Nanobodies are ten times smaller than conventional antibodies (15 kDa compared to 150 kDa), which allows them to penetrate the tumor effectively and clear more quickly from the body when not associated with their target [13,14]. Moreover, low immunogenicity potential and high solubility make them an ideal targeting moiety for targeted therapies [15]. In our previous studies, EGFR [16], c-Met [17], and US28 [18] targeted nanobodies conjugated to the photosensitizer IRDye700DX showed specific and potent cytotoxic effects on cells overexpressing these targets. As a proof of principle study, nanobody-targeted PDT was applied on an oral squamous cell carcinoma orthotopic mouse tumor model overexpressing EGFR. Light was applied 1 h post injection of the EGFR targeted nanobody–PS conjugates, leading to approximately 90% of tumor necrosis and importantly minimal damage to the surrounding normal tissues [19]. In a more recent study, HER2 targeted nanobody–PS conjugates were injected intravenously in HER2-positive breast cancer orthotopic mouse tumor model. Illumination 2 h later induced significant tumor regression after a single nanobody-targeted PDT treatment [20].

Following up on the promising results we obtained in both in vitro and in vivo studies, we explored the possibility of combined targeting of endothelial and cancer cells in vitro, in order to improve the efficacy of targeted PDT. We hypothesized that dual targeting of receptors on endothelial and cancer cells is likely beneficial in tumors with high heterogeneity of target expression and/or intermediate/low target expression levels [21,22]. In line with this, our previous study showed that dual targeting of two cancer cell targets, namely HER2 and CAIX, improved tumor imaging in an orthotopic model of breast cancer [23]. For VTP, one of the target proteins which is mainly overexpressed on tumor-associated vessels, is vascular endothelial growth factor receptor 2 (VEGFR2). This receptor belongs to the tyrosine kinase family and has been shown to play an essential role in tumor angiogenesis and progression [24]. VEGFR2 is activated upon binding of its natural ligand (VEGF). This initiates a phosphorylation cascade that ultimately results in the proliferation and migration of endothelial cells [25]. Under hypoxic conditions, VEGF is upregulated, leading to the subsequent upregulation of VEGFR2 expression in vascular endothelial cells, which ultimately leads to neovascularization [26,27]. Higher expression of VEGFR2 on tumor-associated vessels and lower expression in normal blood vessels makes this receptor an ideal target for selective delivery of therapeutics to the tumor neovasculature [28].

To enable targeting of endothelial cells with nanobody-targeted PDT, we developed and characterized novel nanobodies targeting mouse VEGFR2. Nanobodies were conjugated to IRDye700DX and the phototoxicity induced by the conjugates was assessed in vitro on murine endothelial cell lines with different levels of VEGFR2 expression in monocultures. For simultaneous targeting of endothelial and cancer cells, a previously described EGFR targeted nanobody–PS conjugate was employed [16,19] and in co-cultures an oral squamous cell carcinoma cell line was combined with a murine endothelial cell line. Our results confirm the hypothesis that dual targeting of endothelial and cancer cells leads to more potent PDT. 

## 2. Results

### 2.1. Generation of High Affinity Anti-VEGFR2 Nanobodies

**Figure 1:**
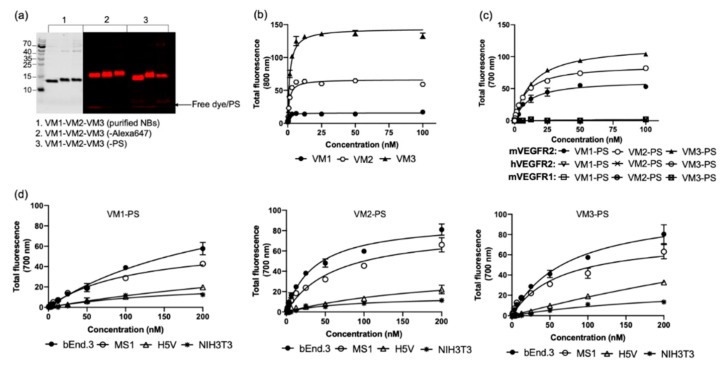

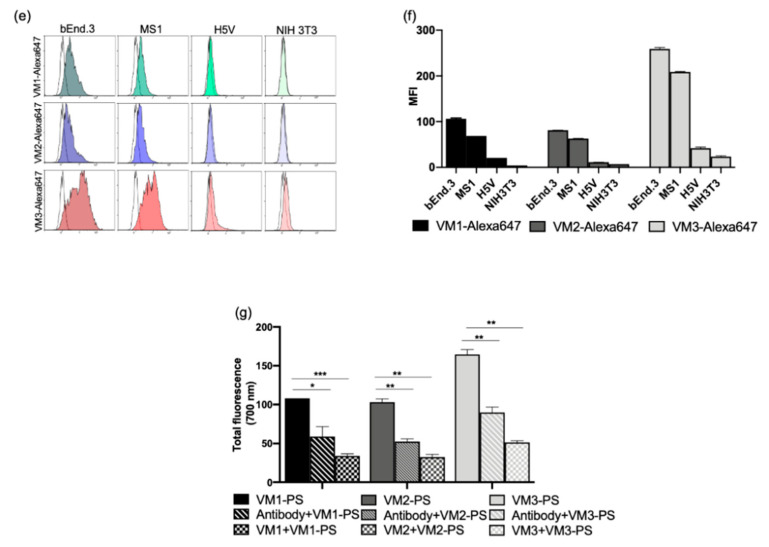
Purity and specificity of the NBs and NB–conjugates. (**a**) Purified NBs, NB–Alexa647, and NB–PS conjugates separated by SDS-PAGE. Free dye/PS is observed at the gel front (arrow): (1) purified NBs after PageBlue staining (depicted in black); and (2,3) the fluorescence of Alexa647 and PS detected at 700 nm, respectively (depicted in red). (**b**) Binding of the unlabeled NBs to the mVEGFR2 protein detected by anti-VHH antibody. (**c**) Binding of the NB–PS conjugates to the mVEGFR2, hVEGFR2, and mVEGFR1 proteins. Total fluorescence of NB–PS bound to the protein was detected using an Odyssey infrared scanner at 700 nm. (**d**) Total fluorescence intensity of cell bound/internalized NB–PS conjugates on the murine cell lines after 1-h incubation at 37 °C. (**e**,**f**) Fluorescence of NB–Alexa647 conjugates detected by flow cytometry. The murine cell lines were incubated with the conjugates for 1 h at 37 °C followed by trypsinization and FACS analysis. Mean fluorescent intensity (MFI) obtained from flow cytometry. Data are shown as mean ± SD. (**g**) In vitro competition experiments of the NB–PS conjugates tested on bEnd.3 cells in the presence or absence of ten-times excess of anti-VEGFR2 antibody or unconjugated NBs. Total fluorescence intensity of the associated NB–PS conjugates was detected by an Odyssey infrared scanner at 700 nm (* *p* < 0.05; ** *p* < 0.01; *** *p* < 0.001; *t*-test).

To obtain anti-VEGFR2 nanobodies, two llamas were injected with mouse VEGFR2 (mVEGFR2) protein and bEnd.3 cell-derived vesicles, followed by construction of the phage libraries as described in Section 4. Upon panning, 96 clones were screened for specific binding to VEGFR2 by means of a phage ELISA. Over 50 clones showed specific binding to VEGFR2 protein and after sequencing, ten nanobodies with unique sequences were selected for further characterization. After preliminary tests, seven nanobodies were excluded due to the low binding affinity to the target protein and the study was continued with the three most promising nanobodies, named VM1, VM2, and VM3. The nanobodies were produced in bacteria using a bioreactor and affinity purified from the periplasmic fractions with Äkta Xpress chromatography. The yield of production for each nanobody was approximately 60 mg of nanobody from 5 L of culture, and all nanobodies were obtained with excellent purity (Figure 1a-1). To determine binding affinity of the nanobodies to the target protein, binding assays were performed on the mVEGFR2 protein and the results indicate that all three nanobodies bound with high affinity to the target protein (Figure 1b). The apparent binding affinity of the nanobodies was 0.8 ± 0.1 nM for VM1, 1.2 ± 0.2 nM for VM2, and 1.5 ± 0.1 nM for VM3.

### 2.2. Nanobodies Remain High Affinity Binders after Random Conjugation

**Figure 2:**
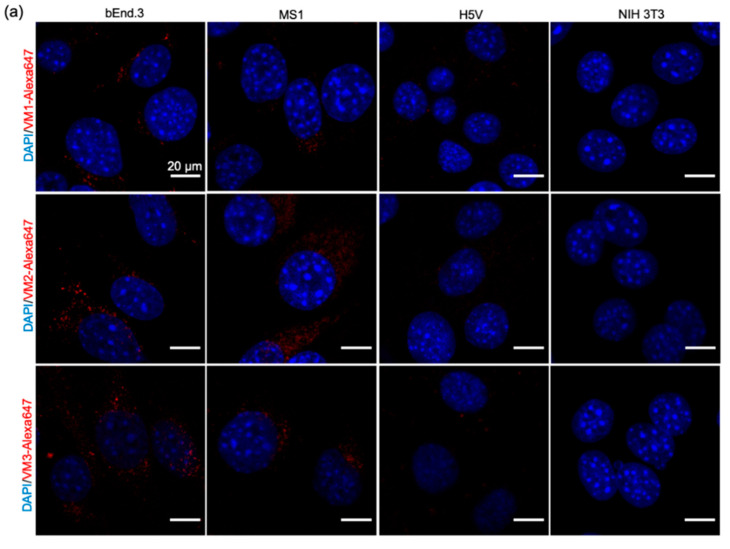

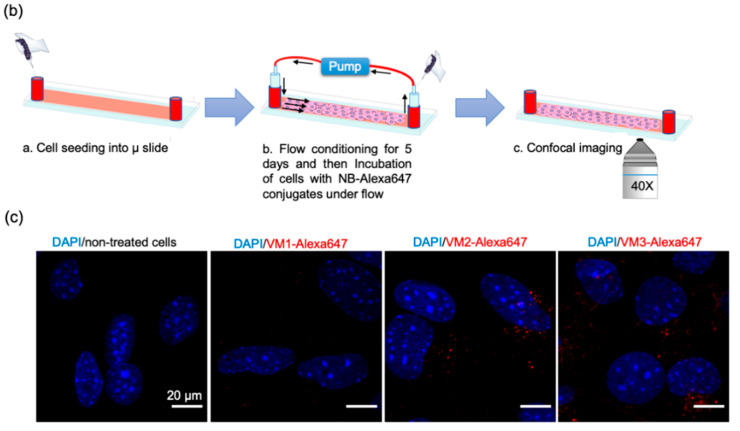
Association of the NB–Alexa647 conjugates with the murine cells after 1-h incubation under static and flow conditions. (**a**) Confocal images of the cells incubated with the NB–Alexa647 conjugates for 1 h at 37 °C. Scale bar: 20 μm. (**b**) Schematic representation of the experimental setup for the flow study using ibidi pump system^®^. bEnd.3 cells were seeded into the μ slides on Day 1 and kept under a unidirectional flow for five days. Cells were incubated with the NB–Alexa647 conjugates for 1 h under flow, and slides were subsequently prepared for confocal imaging. (**c**) Representative images obtained with a confocal microscope after incubation of the conjugates with bEnd.3 cells under flow (shear stress 300/s). Nuclei stained in blue and the conjugates shown in red. Scale bar: 20 μm.

**Figure 3:**
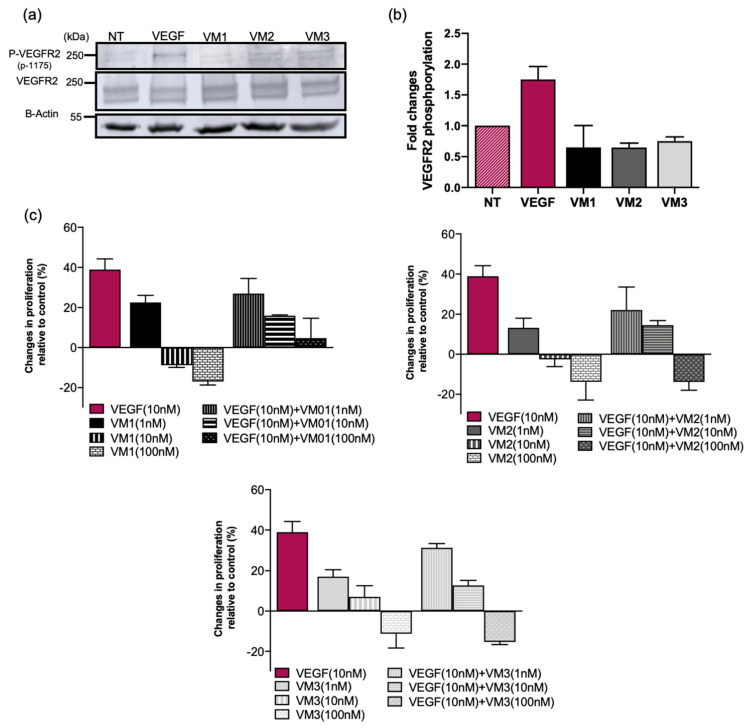
Anti-VEGFR2 NBs blocked VEGF-induced proliferation and did not act as receptor agonists. (**a**) VEGF-A (50 nM) or NBs (1 µM) were added to the serum-starved MS1 cells and incubated for 15 min. VEGFR2 phosphorylation was measured in the total cell lysates by Western blotting: (top) staining of phosphorylated tyrosine 1175 of VEGFR2; (middle) staining of total VEGFR2; and (bottom) staining of actin. (**b**) Fold changes of P-VEGFR2 in MS1 cells treated with VEGF or nanobodies relative to the non-treated cells (NT). (**c**) MS1 cells treated with VEGF-A/NBs or both for 72 h followed by viability assay using AlamarBlue^®^ reagent. Data are presented as percent changes in cell proliferation relative to the non-treated cells (mean ± SD).

As these nanobodies were meant to be used in PDT studies, we first tested the effect of the conjugation strategy on binding properties. For either microscopic or flow cytometry-based characterizations, all nanobodies were conjugated to Alexa647, while for PDT they were conjugated to the PS IRDye700DX. The conjugation of the nanobodies to PS/fluorophore occurred via random NHS-mediated coupling to primary amines in the nanobodies sequence. After purification, less than 2% free fluorophore was detected on SDS-PAGE gel for all nanobodies (Figure 1a-2). As for PS, in all conjugates less than 10% free PS was detected upon conjugate preparation (Figure 1a-3). Determination of the degree of conjugation (DOC) revealed that all nanobodies were on average conjugated to 1 molecule of fluorophore and 1.2 molecules of PS. To verify binding of the conjugates to the target protein, binding assays were performed with all the conjugates on mVEGFR2 protein. The apparent binding affinity of the NB–PS conjugates were: 9 ± 0.8 nM for VM1–PS, 7.4 ± 0.2 nM for VM2–PS, and 11 ± 0.4 nM for VM3–PS (Figure 1c), thus binding affinities remained in the low nM range after conjugation. Similar values were obtained with NB–Alexa647 conjugates. To confirm the specificity of the nanobodies, binding of the NB–PS conjugates was performed on mouse VEGFR1 (mVEGFR1) and human VEGFR2 (hVEGFR2) proteins. The nanobodies did not bind to either mVEGFR1 or hVEGFR2, confirming their specificity towards mVEGFR2 (Figure 1c).

### 2.3. Nanobodies Associate with Murine Cell Lines According to Their VEGFR2 Expression Level

Next to testing conjugates on purified protein, the specificity of the nanobodies was also verified on cells, using different techniques. For these, a panel of murine cell lines was employed, for which the expression of VEGFR2 was assessed using a commercial anti-VEGFR2-PE antibody with flow cytometry. Different levels of VEGFR2 expression were observed in the murine cell lines, i.e., bEnd.3 > MS1 > H5V, while NIH 3T3 are negative for VEGFR2 expression (Figure S1). 

We then investigated the association of the NB–PS conjugates with the cells in 96-well plates. Cells were incubated with different concentrations of the NB–PS conjugates for 1 h at 37 °C and the total fluorescence was plotted against concentration (Figure 1d). A clear correlation was observed between the total fluorescence intensity and the expression level of VEGFR2 on the cells, (i.e., bEnd.3 with the highest and NIH 3T3 with the lowest association) indicating the nanobodies specifically bind VEGFR2. The same trend of association per cell was observed with the NB–Alexa647 conjugates through flow cytometry (Figure 1e,f), which is in agreement with the data obtained with the commercial antibody (Figure S1). Interestingly, although the DOC of all nanobodies was the same, the greater association was observed with VM3, in both assays.

To further verify the specificity of the VEGFR2 nanobodies, bEnd.3 cells were pre-incubated with anti-VEGFR2-PE antibody or unconjugated nanobodies, followed by addition of the NB–PS conjugates. Addition of ten times molar excess of the antibody or unlabeled nanobodies resulted in a significant reduction in the cell-associated NB–PS conjugates, indicating competition of the nanobodies with anti-VEGFR2-PE antibody and with the unconjugated nanobodies (Figure 1g).

### 2.4. Nanobodies Are Internalized after 1 h Incubation under Static and Flow Conditions

To investigate the association of the nanobodies with VEGFR2 on cells (Figure 1d) in more detail, confocal imaging was performed after 1-h incubation at 37 °C under static and flow conditions. As control, the expression/localization of VEGFR2 in the murine cell lines was verified using a commercial antibody (Figure S2). Cells incubated with the antibody showed perinuclear VEGFR2 staining, with varying intensities correlating with the level of VEGFR2 expression (Figure S1), and no signal was observed in the NIH 3T3 cells, which lack VEGFR2 expression. A similar pattern was observed with the NB–Alexa647 conjugates (Figure 2a). All the conjugates showed association with the VEGFR2 expressing cells, while none of them associated with the negative cells (NIH 3T3, Figure 2a).

The interaction of the nanobodies with bEnd.3 cells was investigated after incubation under flow conditions using ibidi pump system^®^ (Figure 2b) to assess whether the nanobodies had sufficient affinity to associate with endothelial cells under flow conditions. Incubation under flow led to similar images of NB–Alexa647 conjugates associated with bEnd.3 cells (Figure 2c). As could be expected from association experiments conducted under flow, the association of nanobodies with cells was less pronounced than in static conditions. Nevertheless, both VM2 and VM3 were clearly taken up by bEnd.3 cells.

### 2.5. Nanobodies Are Non-Agonists and Inhibit VEGF-A Induced Proliferation of MS1 Cells

Incubation of serum-starved MS1 cells with VEGF-A for 15 min at 37 °C showed increased phosphorylation of tyrosine 1175 of VEGFR2 (Figure 3a). Anti-VEGFR2 nanobodies did not stimulate VEGFR2 phosphorylation, indicating that the nanobodies do not act as receptor agonists (Figure 3b). Interestingly, anti-VEGFR2 nanobodies showed antiproliferative effects in the presence or absence of VEGF-A. Treatment of MS1 cells in low serum medium containing 10 nM VEGF-A over a three-day period resulted in about 40% increase in cell proliferation relative to the non-treated cells. In general, nanobodies alone did not stimulate cell proliferation, although a slight proliferation is suggested at the lowest concentration of the nanobodies (1 nM). As this was observed after a three-day incubation, it is likely not relevant in the context of PDT. In addition, these nanobodies inhibited VEGF-stimulated proliferation in a dose dependent manner, which suggests competition of the nanobodies with VEGF-A (Figure 3c). This is in line with the result of the competition assay in which the addition of 10× or 100× excess of VEGF resulted in a significant reduction in the protein-bound NB–PS conjugates (Figure S3).

### 2.6. VEGFR2 Targeted NBs–PS Conjugates Are Potent and Specific PDT Agents

**Figure 4:**
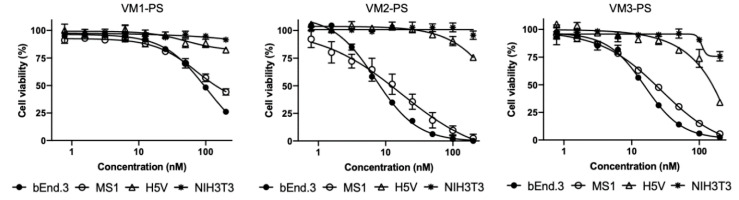
In vitro nanobody-targeted PDT in monoculture. The percentage of cell viability 24 h after illumination relative to the non-treated cells. Cells were incubated with different concentrations of the NB–PS conjugates for 1 h at 37 °C and illuminated with 7 mW/cm^2^ fluence rate and a total light dose of 20 J/cm^2^ using a 690-nm diode laser through a 600-μm optic fiber.

The ability of the NB–PS conjugates to kill VEGFR2 expressing cells was assessed by measuring cell viability one day after illumination of NB–PS associated cells (the association is referred as pulse and described in Figure 1d). The cytotoxicity induced correlated well with the level of VEGFR2 expression: bEnd.3 and MS1 were effectively killed, and, importantly, cells with no VEGFR2 expression were not affected (Figure 4, corresponding EC_50_ values are listed in Table 1). Among the NB–PS conjugates, the strongest cytotoxicity was induced by VM2–PS and VM3–PS.

### 2.7. Cytotoxicity Is Enhanced by Combining the EGFR and VEGFR2 Targeted NB–PS

**Figure 5:**
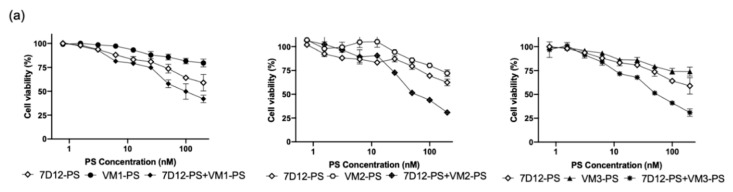

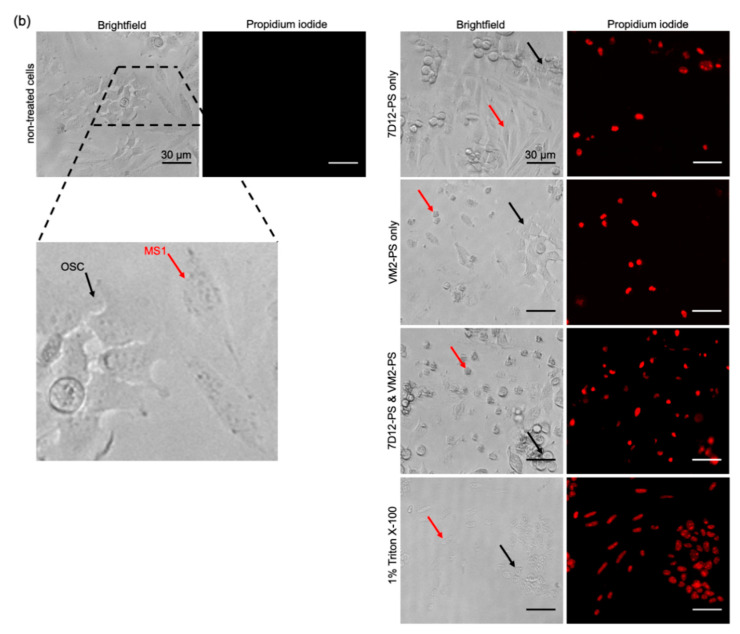
In vitro nanobody-targeted PDT in co-culture of endothelial and cancer cells. (**a**) The percentage of cell viability 24 h after illumination relative to the non-treated cells. Murine endothelial cells (MS1) and human cancer cells (OSC) were co-seeded with 1:3 ratio and incubated with different concentrations of VM–PS conjugates targeting VEGFR2 on endothelial cells, and 7D12–PS targeting EGFR on cancer cells for 1 h at 37 °C, then illuminated with 7 mW/cm^2^ fluence rate and a total light dose of 20 J/cm^2^ using a 690-nm diode laser through a 600-µm optic fiber. (**b**) MS1/OSC co-culture treated with VM2–PS (targeting VEGFR2 on MS1) or 7D12–PS (targeting EGFR on OSC cells) or the mixture of both. Dead cells were stained with propidium iodide 24 h after illumination. MS1 and OSC cells shown with red and black arrows, respectively. Scale bar: 30 μm.

To improve PDT efficacy, the combination of endothelial and cancer cell targeting was explored. For this, co-culture experiments were conducted with the human oral squamous cell carcinoma (OSC) and murine endothelial cells (MS1). MS1/OSC co-cultures were incubated with different concentrations of the VEGFR2 targeted nanobodies, the EGFR targeted nanobody 7D12–PS (described in [16,19]), or their combination. To perform a head-to-head comparison of cytotoxicity induced after PDT, the total concentration of PS conjugate was the same for the combination and the single conjugates (the DOC was used for this correction). When the individual NB–PS conjugates were used to target VEGFR2 or EGFR, we could not obtain low nM EC_50_ values, as we did with the monocultures (Table 1). In the contrary, EC_50_ were above the highest tested concentration. However, when the combination of 7D12–PS and anti-VEGFR2 nanobodies were incubated in the co-culture setup, cellular cytotoxicity significantly increased compared to the individual treatments (Figure 5a, for concentration ≥ 50 nM, * *p* < 0.05 for VM1, and ** *p* < 0.01 for VM2 and VM3, *t* test), with EC_50_ values of 103 ± 1 nM for VM1, 72 nM ± 1.1 for VM2, and 60 nM ± 1.2 for VM3. These results indicate the combination of both conjugates increased effectiveness of nanobody-targeted PDT.

Furthermore, the combination seems to be more potent than the sum of the separate effects: as an example, 100 nM of combined VM2–PS and 7D12–PS (50 nM of each) resulted in 56 ± 0.8% cell death, while 50 nM of 7D12–PS caused 20 ± 2.4% and VM2–PS 14 ± 1.5% cell death (sum would be approximately 35% cell death, * *p* < 0.05). Overall, the results suggest a synergistic cytotoxicity resulting from the combination of the nanobodies, as higher cytotoxicity was obtained with the combined nanobodies compared with the sum of the two separate treatments.

### 2.8. MS1 or OSC Cells Are Specifically Killed in Co-Cultures

The specific cell killing by nanobody-targeted PDT in the co-culture setup was confirmed by staining dead cells with PI, after nanobody-targeted PDT with each single conjugate. In the co-culture setup, MS1 and OSC cells were distinguished by different morphology: MS1 appear as elongated cells (red arrows, Figure 5b), while OSC appear as round cells grown in patches (black arrows, Figure 5b). When MS1/OSC cells were incubated with VM2–PS or 7D12–PS separately, only MS1 or OSC cells were killed, respectively. Treatment of MS1/OSC cells with the combination of VM2–PS and 7D12–PS resulted in more extensive cell cytotoxicity, killing almost every cell (Figure 5b).

## 3. Discussion

Efficacy of targeted PDT can be hampered by heterogeneity of target expression and/or moderate/low target expression levels. To circumvent these, we explored the possibility of combined targeting of endothelial and cancer cells in vitro, as a proof of concept. For this, we developed nanobodies targeting mouse VEGFR2, which is overexpressed mainly on tumor vasculature. After thorough characterization of these nanobodies, we showed that the conjugates are potent PDT agents, specifically killing cells expressing VEGFR2. Moreover, dual targeting of endothelial and cancer cells, using nanobody–photosensitizer conjugates targeting VEGFR2 and EGFR, respectively, showed improved efficacy.

Our mVEGFR2 targeting nanobodies demonstrated high binding affinity to the target protein with single-digit nanomolar dissociation constants (K_D_). Interestingly these nanobodies showed different maximum binding values (B_max_). This might be caused by: (a) the different epitope accessibility of the nanobodies; and/or (b) the indirect recognition of the binding of the nanobodies with anti-VHH antibody, as these nanobodies are from three different sequence families. With directly labeled nanobodies, which remained high affinity binders, the difference in B_max_ was smaller among the different nanobodies, supporting the second explanation. To verify the specificity of the nanobodies, different techniques were applied on both protein and cellular levels. On protein level, we showed that all nanobodies only bound to mVEGFR2 and not hVEGFR2 or mVEGFR1. Among the VEGFR family members, mVEGFR1 was employed because its extracellular domain shares the highest sequence homology (43%) with the one of VEGFR2 [29]. On the cellular level, binding affinity of the nanobodies to the endothelial cells was not determined as endothelial cells detach from the well plates when incubated at 4 °C. However, when conducting the assays at 37 °C, the amount of NB–PS conjugates associated with the murine cell lines correlated well with the expression levels of VEGFR2. This was also verified by flow cytometry analysis, indicating specificity of these nanobodies for mVEGFR2. Other high affinity nanobodies have been described to bind VEGFR2, however only the human variant [30]. The fact that these nanobodies only bind mouse VEGFR2 will enable dedicated studies on the effects of treatment on tumor vasculature. For translational purposes, cross reactive nanobodies could be obtained through dedicated selections.

In our microscopic investigations, internalization and perinuclear accumulation of the NB–Alexa647 conjugates in the cells were observed by confocal imaging, which is in agreement with the results obtained with the commercial anti-VEGFR2 antibody. These results are also in agreement with the findings of other groups where perinuclear staining of VEGFR2 was shown after short incubation with anti-VEGFR2 antibody [31,32]. This perinuclear localization of monovalent nanobodies or antibody is likely a result of constitutive internalization of the receptor, as Gampel and co-workers showed that a significant portion of VEGFR2 undergoes constitutive endocytosis [31]. In nanobody-targeted PDT, the damage is inflicted where the nanobody–PS is located, thus here damage will be mostly perinuclear/intracellular. In addition to the static condition, we also explored the association of the NB–Alexa647 conjugates to the cells under flow condition. In in vivo condition, endothelial cells are exposed to the constant blood flow which might change and reduce the interaction of the nanobodies with the cells. Therefore, we decided to investigate whether incubating bEnd.3 cells with NB–Alexa674 conjugates for 1 h under unidirectional flow would still enable cell association and internalization. The setup we used for the experiments under flow is particularly appropriate to study the interaction of the therapeutics with endothelial cells and can, to some extent, predict the in vivo association of the nanobodies targeting endothelial cells when administered systemically [33]. These experiments were conducted at a shear stress of 300/s, which is comparable to the shear stress reported for the human carotid artery. The alignment of F actin cytoskeleton fibers suggests that cells were shear-adapted (Figure S4), in agreement with other studies [34]. Among the NB–Alexa647 conjugates, VM2 and VM3 were associated with cells. VM1 on the other hand was barely associated with cells, despite showing cell association under the static condition. These results are in agreement with a recently published study showing that the uptake of nanoparticles into endothelial cells is decreased by increasing the flow rate [33,34].

Although nanobody-targeted PDT protocols are conducted in a short period of time, i.e., light is applied 1–2 h post intravenous injection, and involve a single treatment session, we considered it important to assess the agonistic potential of these nanobodies. The phosphorylation assay proved that VEGFR2 is not activated by the nanobodies, ruling out any agonistic activity. The nanobodies did not induce cell proliferation when used at 10 or 100 nM. However, at lower concentration (1 nM), cell proliferation was slightly induced by the nanobodies over the three-day incubation. This likely will not cause any problem in vivo for two reasons: (a) nanobodies have shorter half-life and will be cleared within a day; and (b) the concentration of nanobodies used for in vivo study will likely be higher than 1 nM. Interestingly, the nanobodies also inhibited VEGF-stimulated proliferation in low nanomolar concentrations, suggesting competition of the nanobodies with VEGF (confirmed with a competition assay in Figure S3) and inhibition of VEGF signaling pathways in vitro. However, this inhibition might not be observed in the tumor microenvironment as the concentration and the affinity of the ligand to the receptor is higher than that of nanobodies. Therefore, the competition of the nanobodies with VEGF should be verified in physiological and more complex conditions.

Among the NB–PS conjugates with similar DOC, VM2–PS and VM3–PS induced nearly 100% cell death in bEnd.3 and MS1 cells when tested at 100 nM. EC_50_ values were calculated in nanomolar ranges, indicating that the NB–PS conjugates are potent PDT agents. Importantly, non-VEGFR2 expressing cells were not affected after PDT, providing support for the specificity of nanobody-targeted PDT approach. Besides VEGFR2, there are other receptors on tumor vasculature which can be used for targeted VTP, as recently reviewed [35]. Frochot and colleagues reported integrin αvβ3 targeted peptides for VTP. Phototoxicity induced by cyclic RGD peptide–PS conjugate at 1 μM was assessed after 24-h incubation with HUVEC overexpressing αvβ3 cells and 50% growth inhibition (LD_50_) value was reported 3.1 J/cm^2^ [36]. In another study, Neuropilin-1 targeted heptapeptide was conjugated to tetraphenylchlorin (TPC) and PDT efficacy was determined in HUVEC 24 h after incubation with 100 nM conjugate or free PS. Treatment with free PS showed little cytotoxicity, whereas cells treated with the peptide–PS conjugate had 10.4-fold lower viability. LD_50_ value was reported as 0.47 ± 0.23 and 4.9 ± 0.64 J/cm^2^ for the conjugate and free PS, respectively [37]. Comparison between these studies is difficult due to different conditions employed, however the low nM EC_50_ values presented here certainly encourage further exploration of anti-VEGFR2 conjugates in a preclinical setting.

For the co-culture setup, MS1 cells with moderate level of VEGFR2 expression and OSC cells with moderate level of EGFR expression were selected. In particular for EGFR, our previous research has shown that organoids grown from tumors of patients with head and neck cancers possess low/moderate EGFR levels, compared to cell lines traditionally used in EGFR research (e.g., A431 cell line) [38]. When EGFR and VEGFR2 targeting nanobodies were applied in combination, noting the particular set-up used here with human cancer cells and murine endothelial cells, no interference was observed of either nanobody–PS (VM2–PS or 7D12–PS) on the association of the other nanobody–PS (7D12–PS or VM2–PS) with cells (Figure S5). After illumination, the combined treatment was significantly more effective than the individual treatments Importantly, EC_50_ values were still in nanomolar level when VEGFR2 and EGFR were simultaneously targeted. For the individual treatments, when endothelial and cancer cells were co-seeded, obviously part of cells were not killed due to the lack of target expression, thus the higher EC_50_ values when compared to those obtained in the monoculture setup. Importantly, when nanobodies were applied separately, one cell population was specifically killed (Figure 5b), whereas, when the combination of the nanobodies was used, cytotoxicity was significantly higher. Interestingly, the cytotoxicity is even higher than the sum of the separate effects, which suggests synergism. The higher cytotoxicity induced by combined nanobodies could be due to the overall higher amount of reactive oxygen/nitrogen species produced during illumination, which can affect adjacent cells [1,39]. These results support our hypothesis that simultaneous targeting of endothelial and cancer cells can improve nanobody-targeted efficacy. Follow-up studies will aim at the efficacy evaluation of the conjugates in mice with human tumors. In this context, the cancer cell targeted nanobody–PS conjugates are expected to efficiently distribute through tumors and allow light application 1–2 h after intravenous injection [19,20], while these VEGFR2 nanobody–PS conjugates are expected to interact and be taken up by the (mouse) tumor vasculature. Of note, the time point post injection at which light will be applied will be carefully determined, aiming at the most practical and effective protocol.

Intratumoral heterogeneity is one of the leading causes of therapeutic resistance and treatment failure, and one of the main reasons for poor overall survival in cancer patients. Importantly, it has been shown that targets against which therapeutics are available are not expressed in a homogeneous manner in tumor tissues [21,22,38]. Therefore, combination approaches that target cancer cells and tumor vasculature can potentiate the therapeutic response [40]. Further preclinical studies are needed to investigate the vascular effects of this conjugates and the feasibility for clinical application, to improve selectivity and efficacy of cancer treatment.

## 4. Materials and Methods

### 4.1. Cell Lines

The following murine cell lines were used in this study: brain endothelial cells (bEnd.3), pancreatic islet endothelial cells (MS1), heart endothelial cells (H5V), and fibroblast cells (NIH 3T3). bEnd.3 and H5V cell lines were generous gifts from prof. Enrico Mastrobattista (Department of Pharmaceutical Sciences, Utrecht, The Netherlands) and prof. Ingrid Molema (Department of Pharmacology, Groningen, The Netherlands), respectively. MS1 (ATCC^®^ CRL-2279TM) and NIH 3T3 were (ATCC^®^ CRL-1658™) purchased from ATCC. All cell lines except MS1 were cultured in Dulbecco’s modified eagle’s medium (Lonza, Basel, Switzerland) containing 4.5 g/L glucose and glutamine supplemented with 1% penicillin/streptomycin (Sigma-Aldrich, Zwijndrecht, The Netherlands) and 10% fetal bovine serum (FBS, Sigma-Aldrich). MS1 cells were grown in DMEM from ATCC (30-2002™). Oral squamous cell carcinoma cell line OSC-19-luc2-cGFP (OSC, Radiology and Molecular Imaging department, Leiden, The Netherlands) were cultured as described previously [19].

### 4.2. Llama Immunization and Library Construction

Immunizations of two llamas were performed at Kaneka Eurogentec S.A (Liege, Belgium). Animals received four injections of murine VEGFR2/Fc purified protein (25 μg per injection; R&D Systems, Abingdon, UK) with interval of 2 weeks. As a boost injection, membrane preparations derived from 10^8^ bEnd.3 cells were injected 2 weeks after the final injection [41]. Specific immune response was confirmed by testing pre-immune serum and serum after the second and the final boost. The total mRNA isolated from PBLs was transcribed to cDNA, and specific primers were used to amplify the VhH regions, which were eventually ligated into the phagemid vector pUR8100-Myc-His as described previously [42]. Transformation of electrocompetent TG1 cells resulted in the generation of two libraries of approximately 0.7 × 10^7^ transformants each, which were further used for phage display.

### 4.3. Phase Display Selections and Phage ELISA

Two rounds of phage selections on purified protein were performed in order to identify the specific VEGFR2 binding nanobodies. Phage selection was performed as described previously [42] with the only difference being the amount of coated protein which was 1 μg of VEGFR2/Fc protein or equimolar of an irrelevant protein with Fc tail. For phage ELISA, the protocol described previously was followed [42] except that the amount of coated protein was 300 ng/well VEGFR2/Fc or equimolar of an irrelevant protein with Fc tail.

### 4.4. Nanobody Production and Purification

The nanobody sequences were re-cloned in a modified pET21 to introduce a N-terminal pelB signal sequence and a C-terminal EPEA tag. The resulting plasmids were transformed in heat-shock competent *E. coli* BL21. Single colonies were grown overnight in YT-2x medium supplemented with 100 μg/mL ampicillin and 2% glucose. Nanobodies were produced in a 5-L bioreactor (Eppendorf BioFlo 115 benchtop fermentor) in Terrific Broth (TB) medium containing 0.1% glucose and 100 μg/mL ampicillin and overnight pre-culture (1 mL/100 mL TB). Once bacterial growth was in the log phase, isopropyl β-D-1-thiogalactopyranoside (IPTG) with final concentration of 1 mM was added to induce nanobody expression overnight at 25 °C. The cells were then centrifuged at 4800× *g* for 20 min at 4 °C and the resulting pellet was resuspended in 300 mL PBS. After 2 freeze–thaw cycles, periplasmic fraction was separated by centrifugation at 10,000× *g* for 20 min at 4 °C. The nanobodies were purified by Äkta Xpress chromatography system (GE healthcare, Hoevelaken, The Netherlands) using 5-mL C-tag column (ThermoFisher Scientific, Etten-Leur, The Netherlands) and 5-mL HiTrap Desalting column (GE healthcare). The purity of the nanobodies was verified by SDS-PAGE under reducing condition.

### 4.5. Conjugation of the Nanobodies to Photosensitizer IRDye700DX NHS and Alexa Fluor™ 647 NHS Ester

The nanobodies were conjugated to PS/fluorophore via random NHS-mediated coupling to lysine amino acids, adapted from [16]. Briefly, nanobodies (2 mg/mL) were incubated with 4 molar equivalents of the PS (LI-COR, Bad Homburg, Germany) for 3 h at room temperature (RT). The NB–PS conjugates were purified using four PD-10 desalting columns pre-equilibrated with PBS, as recommended by the manufacturer (GE healthcare). To conjugate nanobodies to Alexa647 NHS Ester (ThermoFisher Scientific), 2 mg/mL nanobodies were incubated with 3 molar equivalents of fluorophore for 2 h at RT. The conjugates were purified from free fluorophore using three Zeba spin desalting columns (2 mL, ThermoFisher Scientific) which were pre-equilibrated with PBS. The purity of the conjugates was determined by SDS-PAGE. Immediately after running the gel, the fluorescence of PS/Alexa647 was detected with an Odyssey infrared scanner at 700 nm. Total protein was subsequently visualized with PageBlue^TM^ staining. The degree of conjugation (DOC) was determined following the manufacturer’s protocol by measuring the absorbance at 280 and 689 nm for PS or 280 and 650 nm for Alexa647 using a Nanodrop spectrophotometer (Nanodrop Technologies, Wilmington, DE, USA). For the co-culture experiment, 7D12–PS conjugate with DOC of 0.5 was prepared as described previously [19].

### 4.6. Determination of Binding Affinity

Mouse VEGFR2 (His tag, Sino Biological, 300 ng/well), equimolar of human VEGFR2 (His tag, Sino Biological, Vienna, Austria) and mouse VEGFR1 (R&D systems) proteins were coated on MaxiSorp^TM^ plate (ThermoFisher Scientific) overnight at 4 °C. Next day, wells were washed twice with PBS and blocked with 4% milk in PBS for 30 min at RT. A dilution range of the nanobodies and NB–PS and NB–Alexa647 conjugates (0.1–100 nM) in 2% mPBS was added to the wells in triplicate and incubated for 2 h at RT. For the labeled nanobodies, after 3 times washing with PBS, fluorescence was detected with an Odyssey infrared scanner at 700 nm. For the unlabeled nanobodies, wells were washed 3 times with 2% mPBS, followed by the addition of rabbit anti-VHH antibody (QVQ B.V.). After 1-h incubation at RT, wells were washed with 2% mPBS and then incubated with IRDye800CW goat anti-rabbit secondary antibody (LI-COR). The plates were scanned the same way as mentioned above. Data were analyzed with GraphPad Prism 8.0 software. The experiments were performed at least three times and data shown as mean ± SD.

### 4.7. Flow Cytometry Analysis

First, four murine cell lines were seeded in 48 well plates (25,000 cells/well, Nunc, Roskilde, Denmark). The next day, cells were incubated with monoclonal anti-VEGFR2-PE antibody (50 nM, ThermoFisher Scientific, catalog n 12-5821-82) for 1 h at 37 °C. Afterwards, cells washed three times with PBS and then trypsinized. The cells were washed once with PBS and then resuspended in 1% BSA-PBS. The same protocol was used for the NB–Alexa647 conjugates (50 nM). The unstained controls for each cell line were taken along. Measurement was carried out on the BD FACSCanto II (BD Biosciences, San Jose, CA, USA) with standard filter sets. Analysis was performed using FlowLogicTM software version 7.3 (Inivai Technologies, Mentone Victoria, Australia).

### 4.8. In Vitro Association without and with Pre-Blocking

To determine association of the NB–PS conjugates with cells, four murine cell lines were seeded (8000 cells/well) in 96-well plates one day before the experiment. Cells were incubated with VEGFR2 targeted NB–PS conjugates (0.78–200 nM) in DMEM medium without phenol red supplemented with 10% FBS, 1% penicillin/streptomycin for 1 h at 37 °C. After 2 times washing with DMEM medium, the total fluorescence of the associated conjugates was measured using an Odyssey infrared scanner at 700 nm.

To explore association of the NB–PS conjugates with cells in the presence of anti-VEGFR2 antibody or unconjugated nanobodies, bEnd3 cells were pre-incubated with 250 nM anti-VEGFR2 antibody (ThermoFisher Scientific) or unconjugated nanobodies for 10 min at RT, followed by the addition of the NB–PS conjugates (25 nM) and incubation for 1 h at 37 °C. NB–PS conjugates were added alone to the cells in order to obtain 100% fluorescent intensity. Cells were washed twice with PBS and total fluorescence was detected at 700 nm. Data were analyzed with GraphPad Prism 8.0 software and shown as mean ± SD. Analysis of significance was performed for each nanobody through unpaired *t*-test. *p* < 0.05 was considered significant.

### 4.9. Immunofluorescence Microscopy

#### 4.9.1. Microscopic Evaluation of the NB–Alexa647 Accumulation in Murine Cell Lines under Static Conditions

Ten thousand cells were seeded a day before the experiment in 16-well Lab-Tek chamber slides (ThermoFisher Scientific). Fifty nanomolar concentration of the NB–Alexa647 conjugates was incubated with the cells for 1 h at 37 °C. Then, cells were washed three times with PBS and fixed with 4% solution of formaldehyde in PBS for 30 min at RT. Formalin-induced autofluorescence was quenched after incubation with 100 mM glycine in PBS for 10 min at RT. Cells were permeabilized with 0.1% Triton X-100 in PBS for 5 min at RT followed by washing with PBS. As control, anti-VEGFR2 monoclonal antibody (55B11, Cell Signaling, Leiden, The Netherlands) was used, following the protocol recommended by the manufacturer. After 1-h incubation with the antibody, wells were washed three times with 0.1% BSA-PBS, and, subsequently, Alexa Fluor™488 Phalloidin (final concentration 33 nM, ThermoFisher Scientific) in 0.1% BSA-PBS was added to the wells and incubated for 30 min. After two times washing with PBS, cells were incubated with 4′,6-diamidino-2-phenylindole (DAPI, 0.25 μg/mL, ThermoFisher Scientific) for 5 min. The slides were mounted with SlowFade (Invitrogen, Leiden, The Netherlands) and imaged with LSM700 confocal laser scanning microscope using 40× oil immersion objective (Carl Zeiss Microscopy, Jena, Germany). The images were analyzed with ImageJ.

#### 4.9.2. Microscopic Evaluation of the NB–Alexa647 Accumulation in bEnd.3 Cells under Flow Conditions

The experiments under flow were performed using ibidi pump system^®^. bEnd.3 cells were seeded in a 0.6-mm μ-Slide I Luer in DMEM medium containing 10% FBS and 1% penicillin/streptomycin at a density of 1.6 × 10^6^ cells per slide. The slides were incubated for 2 h at 37 °C and then connected to a pump-controlled perfusion set under unidirectional and continuous flow (shear rate 300/s, shear stress 0.3 N/m^2^, viscosity 1 mPa·s) at 37 °C in 5% CO_2_ for 5 days before addition of the conjugates. The medium was changed every 2 days. The conjugates diluted in complete medium were added to the system (final concentration 50 nM) while flow was shortly stopped. Cells were incubated with the conjugates for 1 h at 37 °C under flow (shear rate 300/s, shear stress 0.3 N/m^2^, viscosity 1 mPa·s). Afterwards, the flow stopped, the slide was disconnected from the system, and cells were carefully washed three times with PBS. The rest of the cell preparation for confocal microscopy was performed as mentioned above expect for the last step, in which ibidi mounting medium was used before imaging.

### 4.10. Western Blot Analysis of VEGFR2 Phosphorylation

MS1 cells (250,000 cells/well) were seeded in 6-well plates in DMEM medium containing 10% FBS and 1% penicillin/streptomycin and allowed to adhere overnight. The next day, the cells were rinsed with DMEM containing 0.1% FBS and serum-starved overnight in the same medium. The day of the assay, 50 nM VEGF-A (PEPROTECH^®^, Cranbury, NJ, USA) or 1 µM nanobodies were added to the cells in the starvation medium and incubated for 15 min at 37 °C. Afterwards, cells were immediately washed twice with ice-cold PBS and total cell lysates were prepared with scraping cells in 100 µL of 1× Laemmli sample buffer and boiled for 10 min at 100 °C. Proteins were separated on 8% SDS-PAGE and blotted onto PVDF membrane (Roche, Mannheim, Germany). The blots were then blocked with 2% BSA in TBS-T (0.05% Tween-20 in 20 mM Tris-buffered saline pH 7.2, TBS-T) buffer for 1 h at RT, followed by staining overnight at 4 °C for phosphorylated receptor using a rabbit monoclonal anti-VEGFR2 phospho-tyrosine 1175 antibody (19A10, Cell Signaling) diluted in 2% BSA in TBS-T. As a loading control, the lower part of the blot was stained for actin overnight with a mouse monoclonal anti-actin antibody (ICN Biomedicals, Irvine, CA, USA). Afterwards, the blots were incubated with IRDye800CW goat anti-rabbit secondary antibody (LI-COR) and IRDye800CW donkey anti-mouse secondary antibody (LI-COR) for 1 h at RT. Bound antibody was visualized with an Odyssey infrared scanner at 800 nm. Afterwards, blots were stripped using 1% SDS and 25 mM glycine pH 2, blocked with 2% BSA and then incubated with rabbit anti-VEGFR2 monoclonal antibody (55B11, Cell Signaling) overnight at 4 °C. After incubation with IRDye800CW goat anti-rabbit secondary antibody, the total VEGFR2 was visualized at 800 nm.

### 4.11. Proliferation Assay

For the proliferation assay, MS1 cells were used as they showed better compatibility with the assay conditions among the different endothelial cell lines. Two thousand MS1 cells were seeded in 96-well plates in complete medium containing 10% FBS and 1% penicillin/streptomycin. The next day, 10 nM VEGF-A, different concentrations of the nanobodies (1, 10, and 100 nM), or the mixture of both in DMEM containing 0.2% FBS were added to the cells and incubated for 72 h at 37 °C. Cell viability was determined using AlamarBlue^®^ reagent following the manufacturer’s protocol (Bio-Rad, Lunteren, The Netherlands). The percentage of changes in proliferation was calculated relative to the non-treated cells. The experiment was performed at least three times. The assay results are shown as mean ± SD.

### 4.12. In Vitro PDT

#### 4.12.1. Monoculture

Total fluorescence of the associated NB–PS conjugates with the murine cell lines was determined 1 h after incubation at 37 °C, as described in Section 4.8. Immediately after, cells were illuminated with 7 mW/cm^2^ fluence rate for a total light dose of 20 J/cm^2^ using a 690-nm diode laser through a 600-μm optic fiber (Modulight, Tampere, Finland). After overnight incubation at 37 °C, AlamarBlue^®^ reagent was added to the wells following the manufacturer’s protocol (Bio-Rad) to assess cell viability. Cells with no light/no treatment were used as 100% viability and the percentage of viability calculated relative to the 100% viable cells. Data were analyzed with GraphPad Prism 8.0 software and presented as mean ± SD.

#### 4.12.2. Co-Culture of Endothelial and Cancer Cells

MS1 cells were co-seeded with OSC cells in 96-well plates (1:3 ratio and total number of 10,000 cells/well) one day before the experiment. The next day, cells were incubated with different concentrations of 7D12–PS (targeting EGFR on OSC cells), or VEGFR2 targeting conjugates, or the mixture of both keeping the total concentrations of PS the same (e.g., 100 nM 7D12, 100 nM VM, or 50 nM 7D12 + 50 nM VM). After 1 h of incubation at 37 °C, cells were illuminated, and the percentage of cell viability was determined the next day, as described above.

### 4.13. Specificity of the Nanobody-Targeted PDT in Co-Culture Setup

MS1/OSC cells were treated with the NB–PS conjugates (100 nM 7D12–PS, VM2–PS, or 50 nM+ 50 nM PS of mixture), and, 24 h after illumination, the medium was slowly removed, and PI (1 μg/mL, Invitrogen) in PBS was added to the cells and incubated for 10 min at 37 °C. Triton (1%) was used as a positive control to induce 100% cell death. The cells were imaged with an EVOS microscope and analyzed with ImageJ.

## 5. Conclusions

We report the successful development of nanobodies targeting mouse VEGFR2, which demonstrated high association with cells under flow and static conditions. In addition, the nanobody–photosensitizer conjugates were shown to be potent and specific PDT agents. Importantly, we improved the efficacy of nanobody-targeted PDT in co-culture of endothelial and cancer cells when treating the cells with dual targeted nanobody–photosensitizer conjugates. To our knowledge, this is the first time nanobodies targeting mouse VEGFR2 were developed with the potential to be applied for preclinical validation of VTP. Further in vivo studies are needed to verify the synergistic/additive effects of the dual targeted PDT.

## Figures and Tables

**Table 1:** EC_50_ values obtained from nanobody-targeted PDT of two murine endothelial cell lines.

NB–PS	bEnd.3 EC_50_ ± SD (nM)	MS1 EC_50_ ± SD (nM)
VM1	92 ± 1	155 ± 1.1
VM2	8.6 ± 1	11 ± 1.1
VM3	14 ± 1	21 ± 1

## Supplementary Materials

The following are available online at https://www.mdpi.com/2072-6694/12/10/2732/s1, Figure S1: VEGFR2 expression in the murine cell line measured by flow cytometry, Figure S2: Microscopic images of VEGFR2 expression in the murine cell lines, Figure S3: In vitro competition experiment of the NB–PS conjugates tested on purified protein in the presence or absence of 10× and 100× excess of VEGF, Figure S4: Comparison of F actin staining in non-treated bEnd.3 cells cultured under static or flow conditions, Figure S5: Association of the VEGFR2 and EGFR targeted nanobodies, separately and in combination, with cells of MS1/OSC co-cultures.
