# Supplementary material for: Dual Targeting of Endothelial and Cancer Cells Potentiates In Vitro Nanobody-Targeted Photodynamic Therapy
Source: Cancers (Basel). 2020 Sep 23;12(10):2732. doi: 10.3390/cancers12102732 (PMC7650791; doi:10.3390/cancers12102732)

Figure 1a

Fluorescence scan before commassie stain

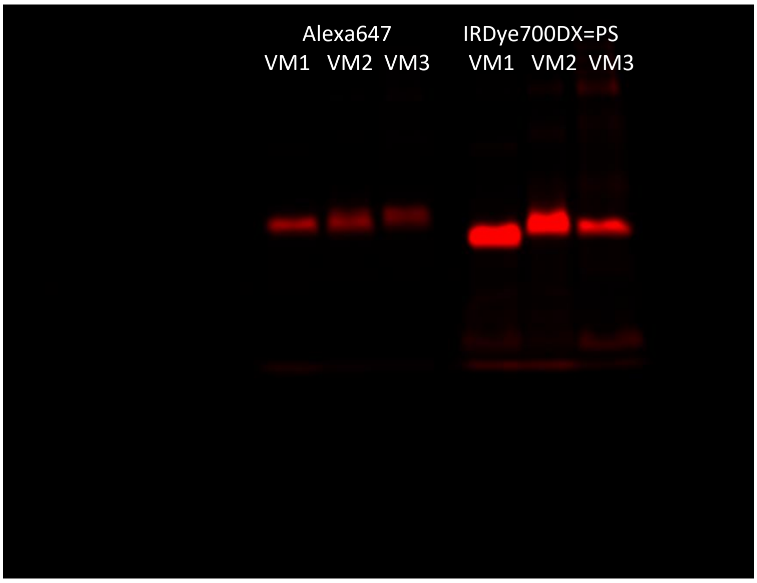

After coomassie stain

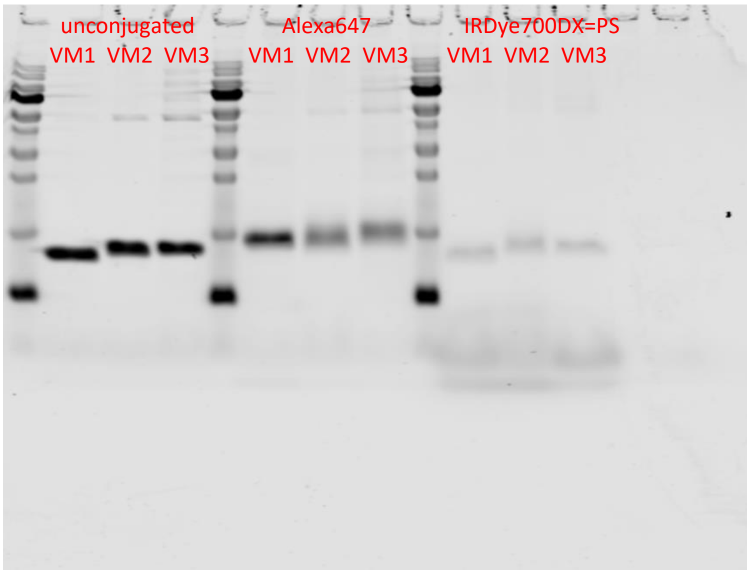

Figure 3a

Phospho VEGFR2

250 kDa

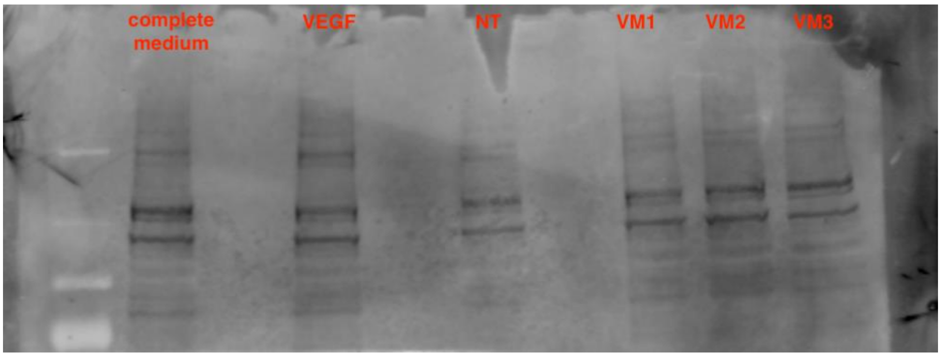

Total VEGFR2

250 kDa

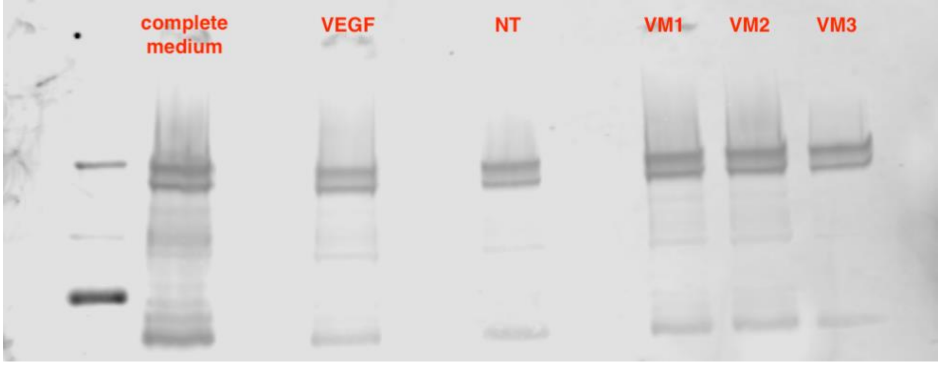

Actin

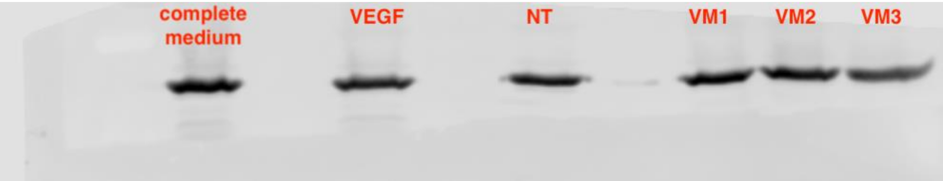

Supplement: Supplementary file 1 [file cancers-12-02732-s001.zip › cancers-916312 uncropped gels blots_marked-1.pdf]
