# Supplementary material for: Dual Targeting of Endothelial and Cancer Cells Potentiates In Vitro Nanobody-Targeted Photodynamic Therapy
Source: Cancers (Basel). 2020 Sep 23;12(10):2732. doi: 10.3390/cancers12102732 (PMC7650791; doi:10.3390/cancers12102732)
Supplement: Supplementary file 1 [file cancers-12-02732-s001.zip › cancers-916312proof╗╪└┤╡─supplementary materials.pdf]

Dual targeting of endothelial and cancer cells potentiates *in vitro* nanobody-targeted photodynamic therapy

Vida Mashayekhi[1], Katerina T. Xenaki[1], Paul van Bergen en Henegouwen[1], Sabrina Oliveira[1,2,*]

[1] Cell Biology, Neurobiology & Biophysics, Department of Biology, Faculty of Science, Utrecht University, 3584 CH, Utrecht, The Netherlands

[2] Pharmaceutics, Department of Pharmaceutical Sciences, Faculty of Science, Utrecht University, 3584 CG Utrecht, The Netherlands

***** Correspondence: s.oliveira@uu.nl; Tel.: +31 634103460

Supplementary Data

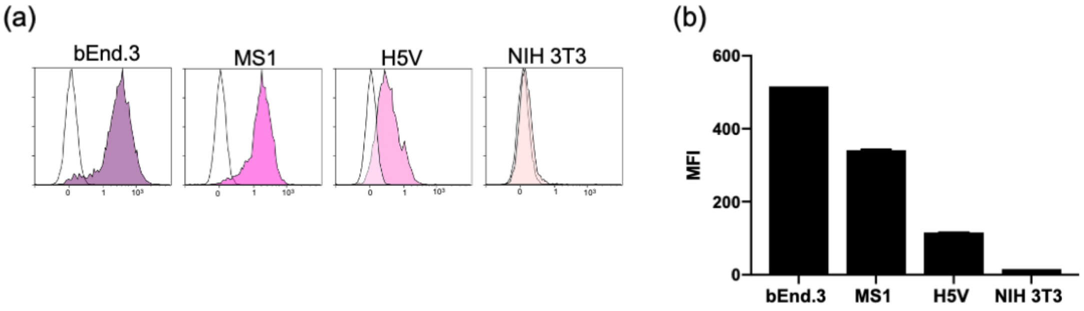

Figure S1. VEGFR2 expression in the murine cell line measured by flow cytometry. **(a)** Fluorescence of VEGFR2 detected by anti-VEGFR2-PE antibody correlates with the level of VEGFR2 expression: bEnd.3> MS1> H5V> NIH 3T3. The murine cell lines were incubated with the antibody for 1 h at 37 °C followed by trypsinization and FACS analysis. **(b)** Mean fluorescent intensity (MFI) obtained from flow cytometry. Data shown as mean ± SD.

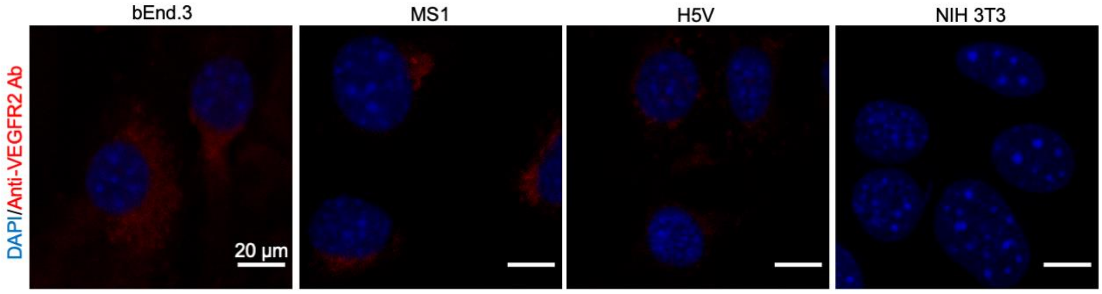

Figure S2. Microscopic images of VEGFR2 expression in the murine cell line. Fluorescence of VEGFR2 detected by anti-VEGFR2 antibody showed perinuclear VEGFR2 staining with varying intensities correlating with the level of VEGFR2, and no signal was observed in the NIH 3T3 cells.

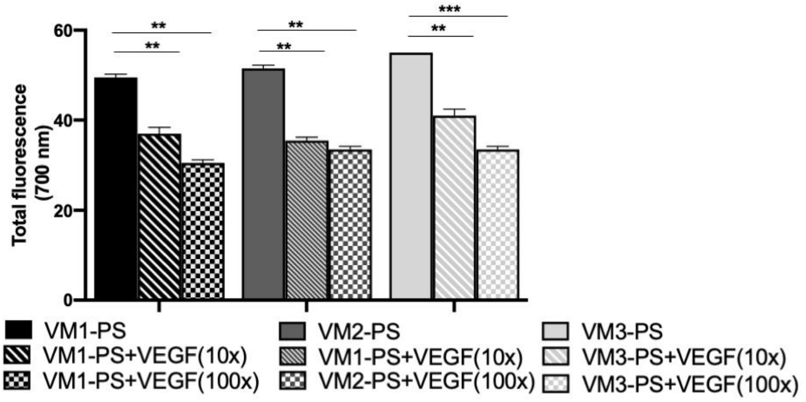

Figure S3. *In vitro* competition experiment of the NB-PS conjugates tested on purified protein in the presence or absence of 10x and 100x excess of VEGF. NB-PS conjugates alone (final concentration 25 nM) or in combination with VEGF (final concentrations 250 nM or 2.5 µM) were added to the mVEGFR2 protein and incubated for 2 h at RT. After 3 times washing with PBS, total fluorescence intensity of the bound NB-PS conjugates was detected by an Odyssey infrared scanner at 700 nm (** $p < 0.01$; *** $p < 0.001$; t test).

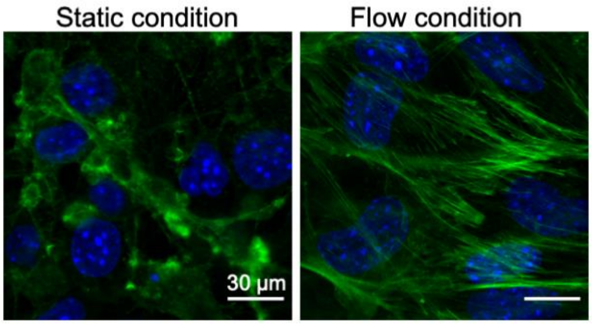

Figure S4. Comparison of F actin staining in non-treated bEnd.3 cells cultured under static or flow conditions. The F actin cytoskeleton staining shows a clear difference between the two conditions. A clear alignment of actin fibers is observed when cells were kept under flow.

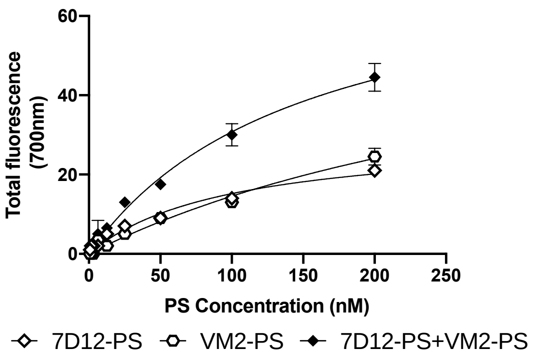

Figure S5. Association of the VEGFR2 and EGFR targeted nanobodies, separately and in combination, with cells of MS1/OSC co-cultures. After 1 h incubation of cells with the conjugates at 37 °C, total fluorescence intensity of the associated NB-PS conjugates was detected by an Odyssey infrared scanner at 700 nm.
